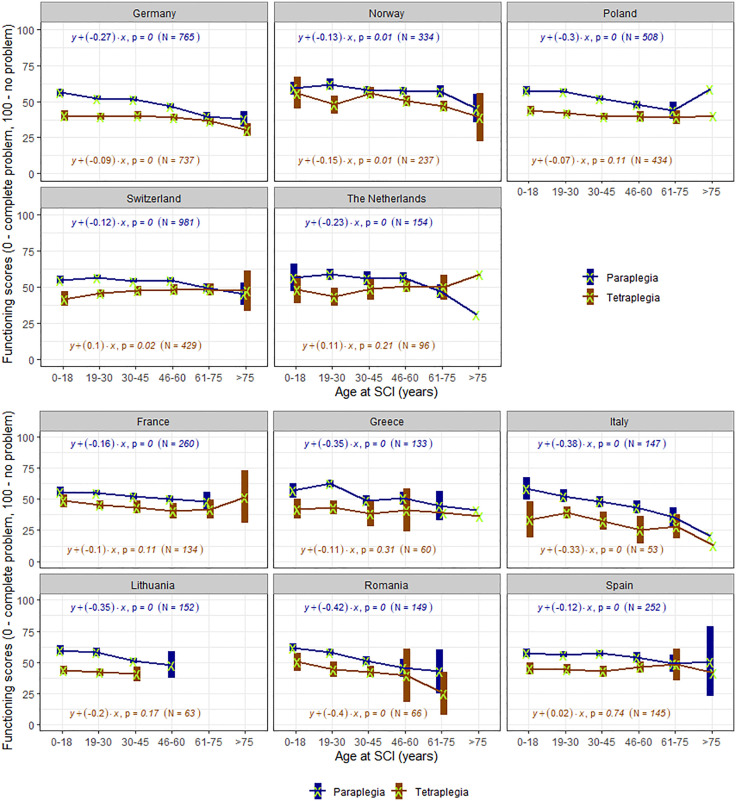# Correction: Ageing, functioning patterns and their environmental determinants in the spinal cord injury (SCI) population: a comparative analysis across eleven European countries implementing the International Spinal Cord Injury Community Survey

**DOI:** 10.1371/journal.pone.0337486

**Published:** 2025-11-25

**Authors:** Carla Sabariego, Cristina Ehrmann, Jerome Bickenbach, Diana Pacheco Barzallo, Annelie Schedin Leiulfsrud, Vegard Strøm, Rutger Osterthun, Piotr Tederko, Vanessa Seijas, Inge Eriks-Hoogland, Marc Le Fort, Miguel A. Gonzalez Viejo, Andrea Bökel, Daiana Popa, Yannis Dionyssiotis, Alessio Baricich, Alvydas Juocevicius, Paolo Amico, Gerold Stucki

## Notice of Republication

This article was republished on November 11, 2025, because Fig 3 appeared incorrectly. It is shown correctly here.

**Fig 3:**