# Correction: Ageing, functioning patterns and their environmental determinants in the spinal cord injury (SCI) population: a comparative analysis across eleven European countries implementing the International Spinal Cord Injury Community Survey

**DOI:** 10.1371/journal.pone.0337859

**Published:** 2025-12-02

**Authors:** The PLOS One Staff

## Notice of Republication

This article was republished September 30. Fig 3 was published incorrectly. Attached is the correct figure.

## Supporting information

S1 File Originally published, uncorrected article. (PDF)

S2 File Republished, corrected article. (PDF)
