# Supplementary figures and images for: Ageing, functioning patterns and their environmental determinants in the spinal cord injury (SCI) population: A comparative analysis across eleven European countries implementing the International Spinal Cord Injury Community Survey
Source: PLoS One. 2023 Apr 20;18(4):e0284420. doi: 10.1371/journal.pone.0284420 (PMC10118153; doi:10.1371/journal.pone.0284420)

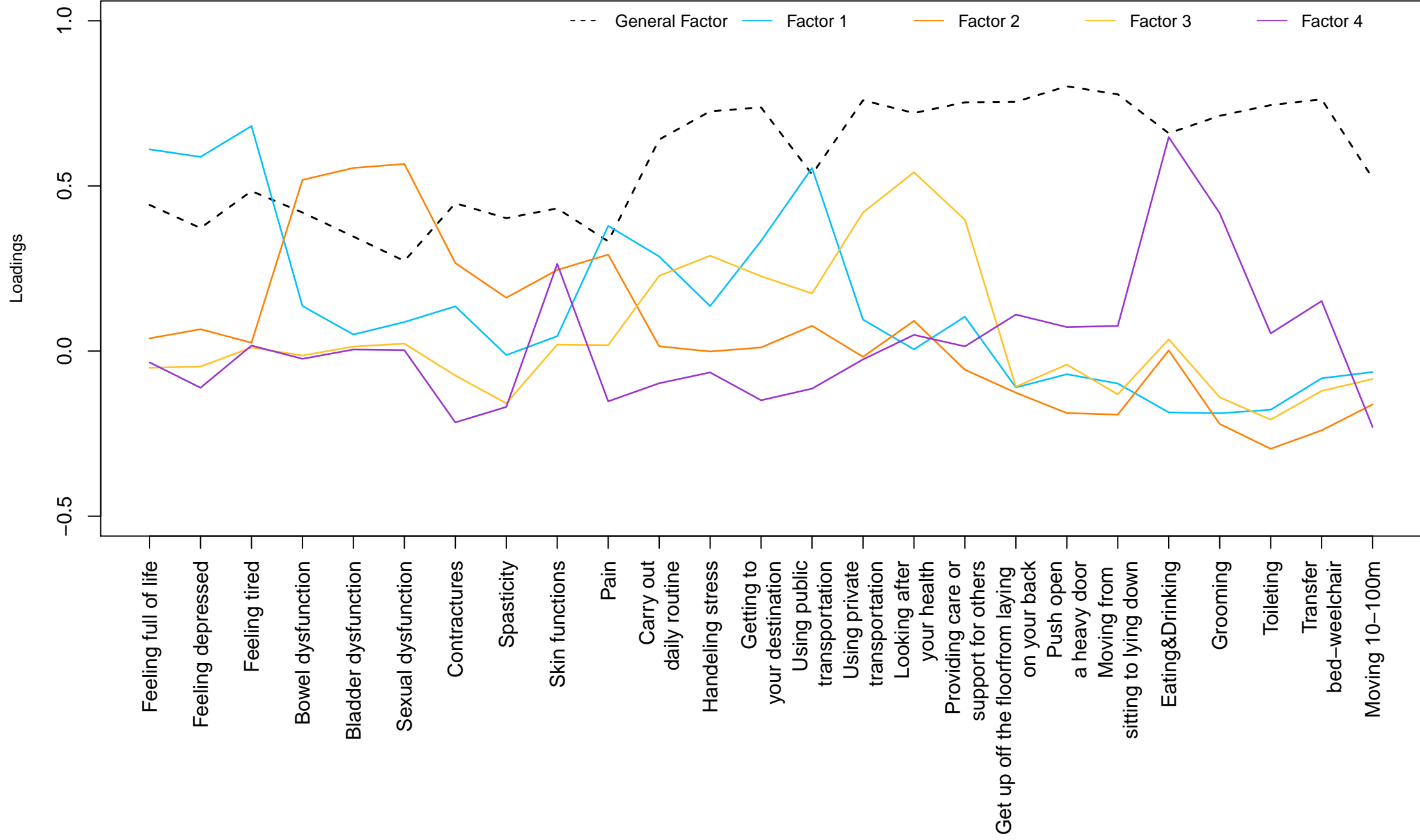

Supplement: S1 Fig — (PDF) [file pone.0284420.s001.pdf]

**Germany (N=1265), R2=44.69%**

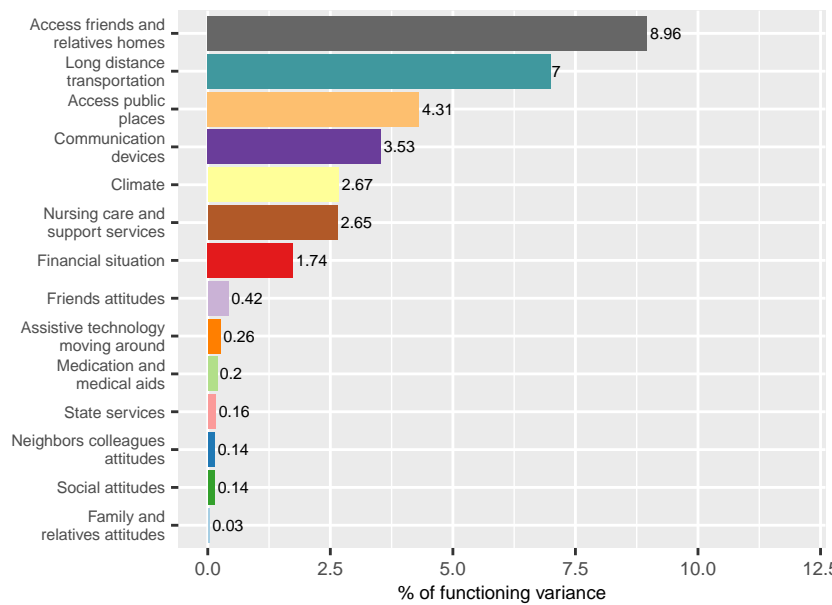

**Switzerland (N=1346), R2=44.71%**

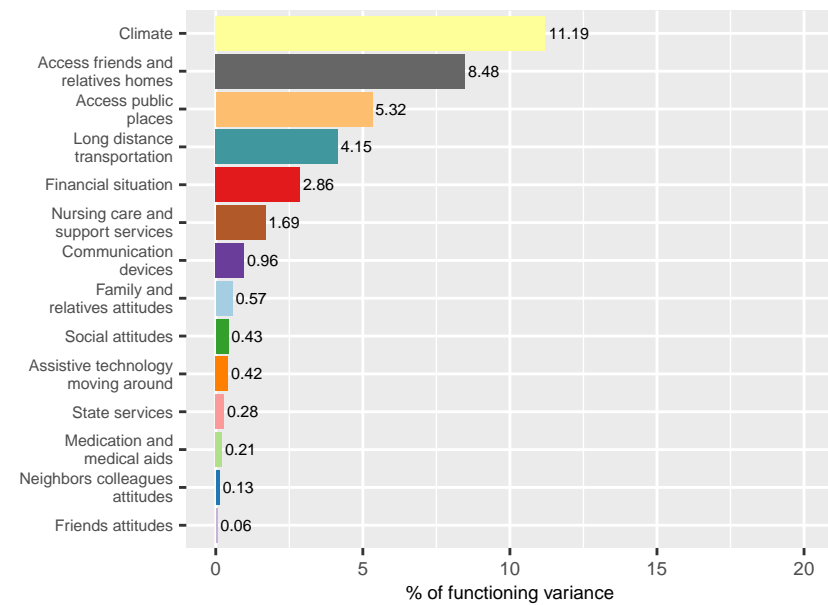

**Poland (N=880), R2=44.69%**

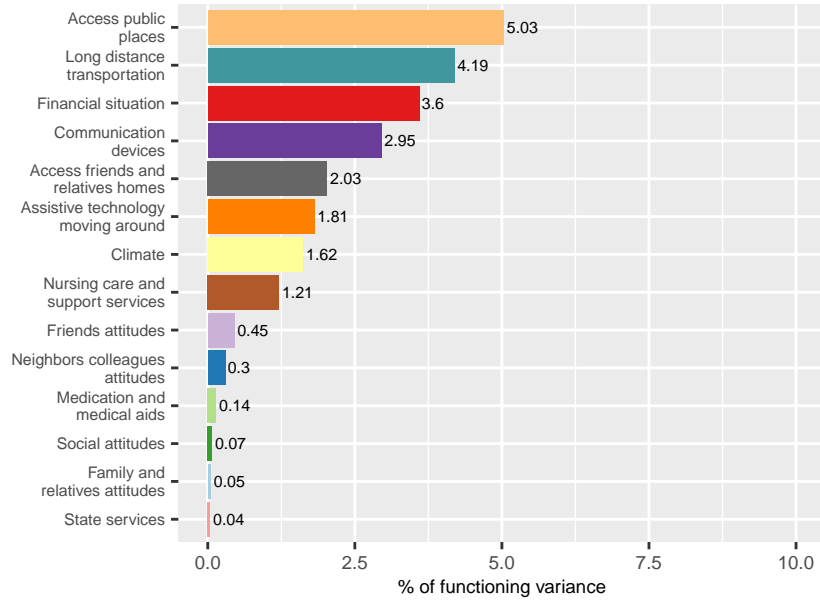

**The Netherlands (N=242), R2=54.42%**

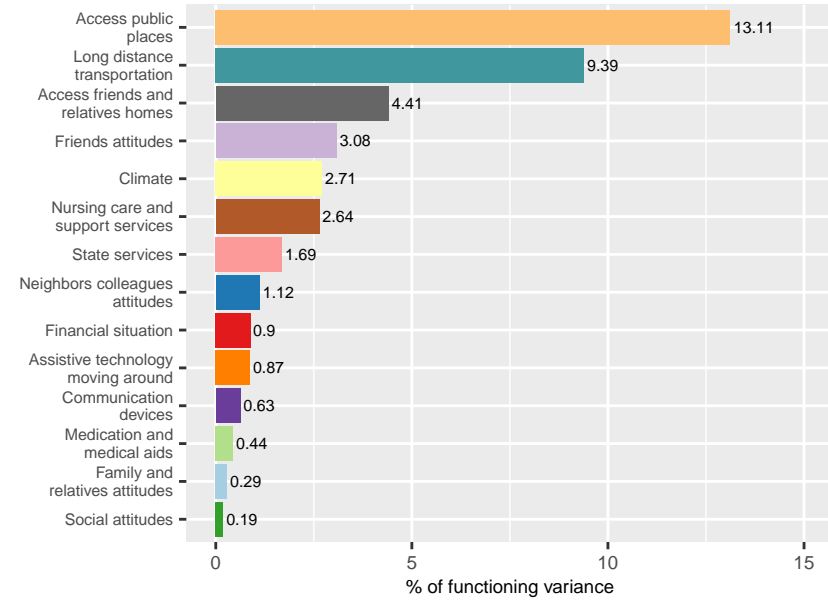

**Norway (N=541), R2=53.88%**

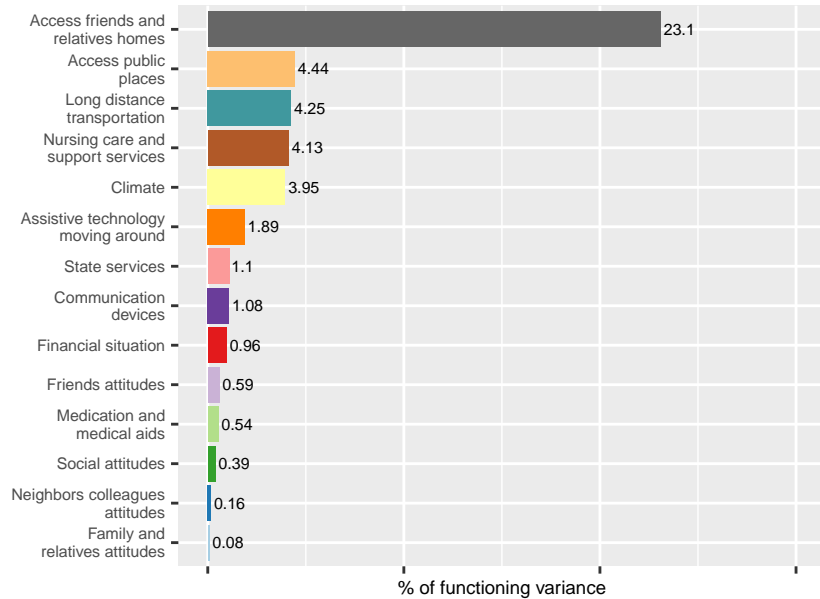

Supplement: S2 Fig — Complete cases for all predictors were considered. For each country, the full model variation when considering EFs and chronological age and type since injury as predictors is indicated in each country figure’s title. (PDF) [file pone.0284420.s002.pdf]

**Spain (N=350), R2=40.41%**

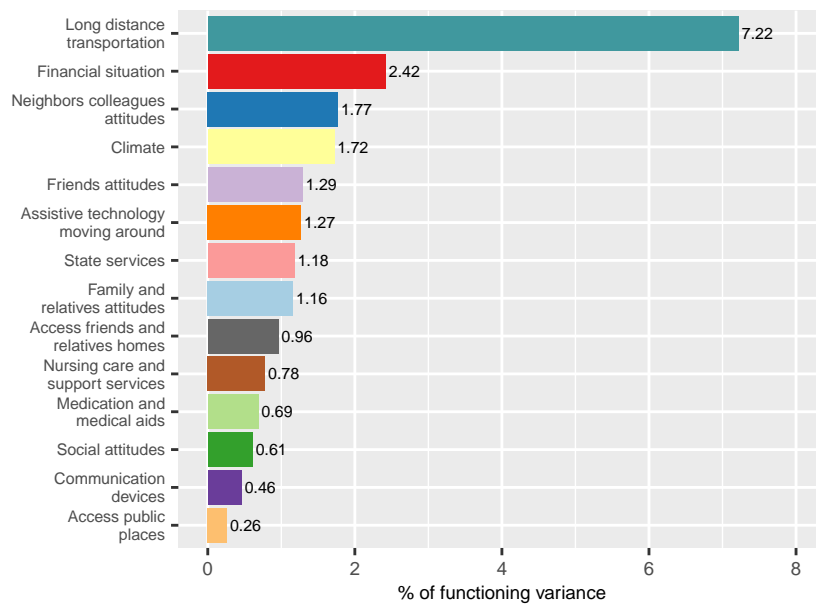

**France (N=350), R2=47.08%**

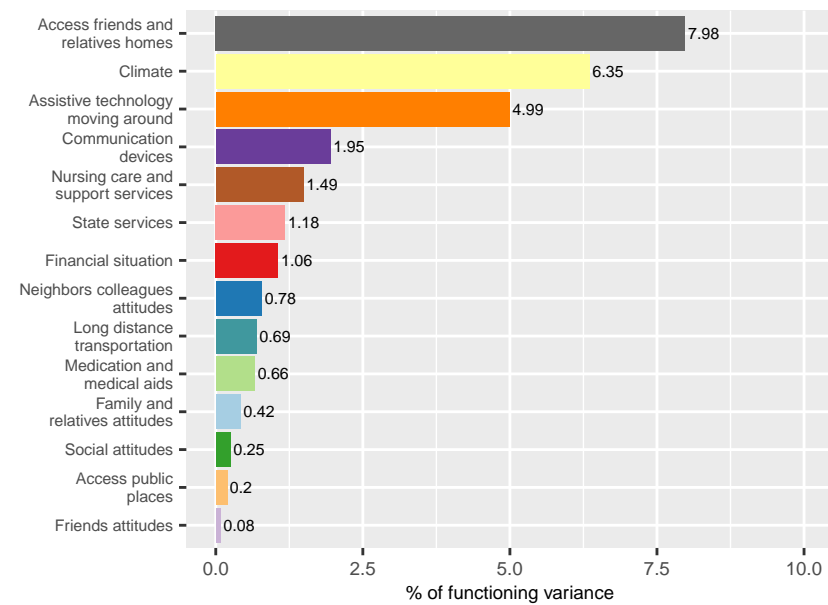

**Lithuania (N=212), R2=75.5%**

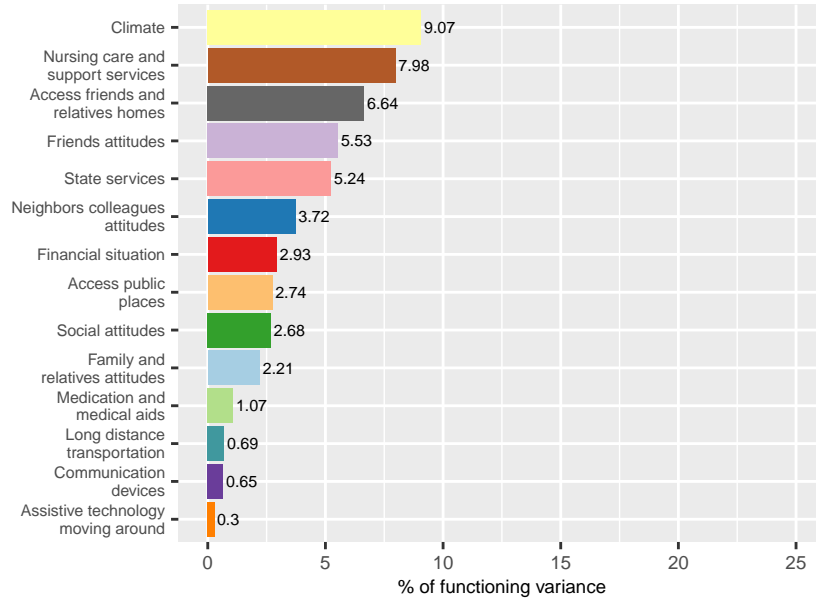

**Romania (N=201), R2=55%**

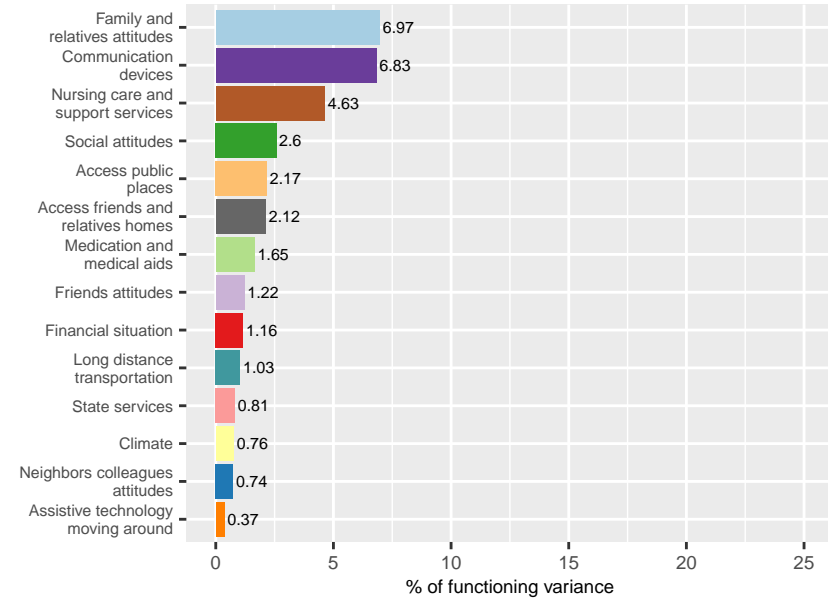

**Italy (N=186), R2=69.99%**

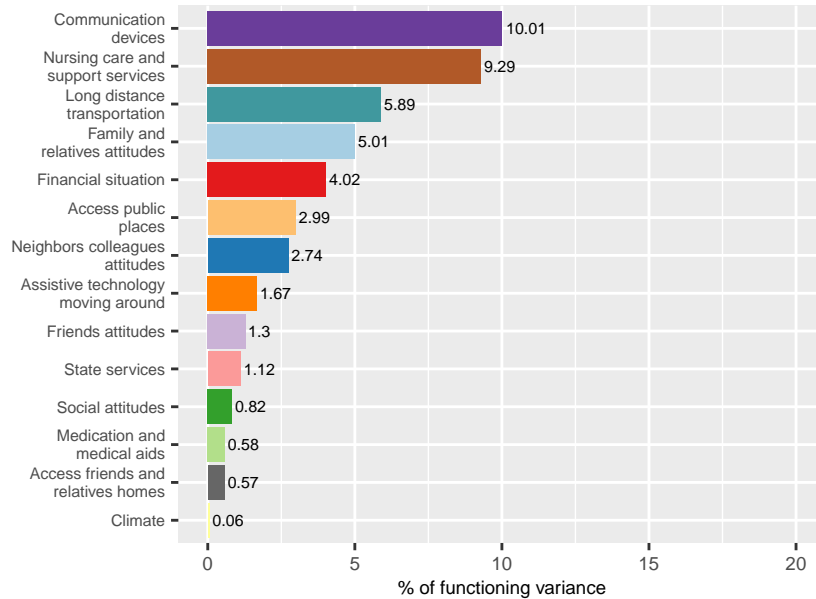

**Greece (N=176), R2=68.32%**

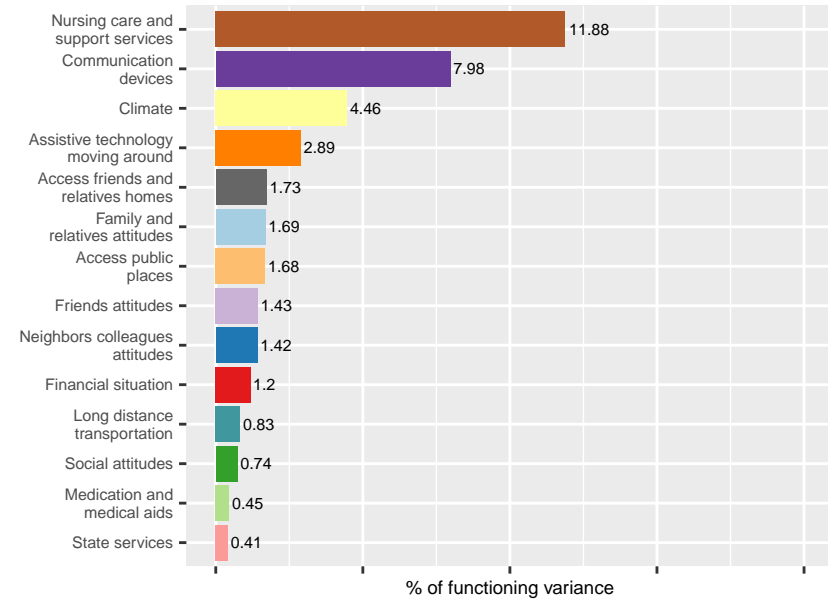

Supplement: S3 Fig — Complete cases for all predictors were considered. For each country, the full model variation when considering EFs and chronological age and type since injury as predictors is indicated in each country figure’s title. (PDF) [file pone.0284420.s003.pdf]
