# Supplementary material for: Ageing, functioning patterns and their environmental determinants in the spinal cord injury (SCI) population: A comparative analysis across eleven European countries implementing the International Spinal Cord Injury Community Survey
Source: PLoS One. 2023 Apr 20;18(4):e0284420. doi: 10.1371/journal.pone.0284420 (PMC10118153; doi:10.1371/journal.pone.0284420)
Supplement: S1 Table — (DOCX) [file pone.0284420.s004.docx]

**S1 Table. Ethics committees or review boards approvals in the 11 International Spinal Cord Injury (InSCI) Community Survey countries.**

| **Country** | **Name of ethics committee or institutional board responsible for ethics approval of the InSCI study** | **Approval number** | **Approval date** | **Form of consent** |
| --- | --- | --- | --- | --- |
| France | Comité de Protection des Personnes | Ref : 180304 | April 10 2018 | A |
| Germany | Ethic Committee of Hannover Medical School | 7374 | Feb 13 2017 | B |
| Greece | Scientific/Ethical Committee of General Hospital 'G. Gennimatas' Athens | 20257/1.8.2016 | Aug 1 2016 | A, B |
| Italy | Comitato Etico Interaziendale AOU 'Maggiore della Carità' di Novara, ASL BI, ASL NO, ASL VCO | ItaSCI, 1, 25-01-2018 | April 27 2018 | A |
| Lithuania | Vilnius Regional Committee for the Ethics of Biomedical Research | 158200-17-907-421 | Mai 9 2017 | A, B |
| Netherlands | Medical Ethics Board University Medical Center Utrecht | WAG/mb/17/024763 | Aug 16 2017 | A |
| Norway | Regional Committee for Medical and Health Research Ethics, South East | 2016/1184/REK sør-øst | Sept 21 2016 | A, B |
| Poland | Bioethical Committee of the Medical University of Lodz | RNN/198/16/KE | July 12 2016 | A, B |
| Romania | Ethical Committee of Rehabilitation Hospital Felix Spa | 2228/06.03.2017 | March 3 2017 | A, B |
| Spain | Ethical Committee of Hospital Universitari Vall d’Hebron, Hospital Universitario de Cruces, Hospital Universitario Materno Infantile de Gran Canaria, Hospital Universitario Virgen del Rocio | PR(ATR)285/2016 | Oct 2016 | A |
| Switzerland | Ethical Committee of Northern and Central Switzerland | 11042 PB_2016-02608 | Dec 21 2016 | A, B |
| Note: A=written consent; B=questionnaire completion considered as implicit consent; | | | | |
