# Supplementary material for: Ageing, functioning patterns and their environmental determinants in the spinal cord injury (SCI) population: A comparative analysis across eleven European countries implementing the International Spinal Cord Injury Community Survey
Source: PLoS One. 2023 Apr 20;18(4):e0284420. doi: 10.1371/journal.pone.0284420 (PMC10118153; doi:10.1371/journal.pone.0284420)
Supplement: S2 Table — (DOCX) [file pone.0284420.s005.docx]

**S2 Table. InSCI questions used in the development of functioning scores.**

| **Selection Criterion** | **ICF category** | **Name of the ICF category** | **Questionnaire Source** | **InSCI - Question** |
| --- | --- | --- | --- | --- |
| ICF Brief Core Set | b152 | Emotional functions | SF-36 V2 Vitality Subscale* | Have you felt downhearted and depressed? |
| ICF category | b130 | Energy and drive functions | SF-36 V2 Vitality Subscale* | Did you feel tired? |
|  |  |  | SF-36 V2 Vitality Subscale* | Did you feel full of life? |
| ICF Brief Core Set | b280 | Sensation of pain | SCI-SCS** | For the following health problems please rate how much of a problem it was for you in the last 3 months: Pain |
| ICF Brief Core Set | b525 | Defecation functions | SCI-SCS** | For the following health problems please rate how much of a problem it was for you in the last 3 months: Bowel dysfunction |
| ICF Brief Core Set | b620 | Urination functions | SCI-SCS** | For the following health problems please rate how much of a problem it was for you in the last 3 months: Bladder dysfunction |
| ICF Brief Core Set | b640 | Sexual functions | SCI-SCS** | For the following health problems please rate how much of a problem it was for you in the last 3 months: Sexual dysfunction |
| ICF Brief Core Set | b710 | Mobility of joint functions | SCI-SCS** | For the following health problems please rate how much of a problem it was for you in the last 3 months: Contractures |
| ICF Brief Core Set | b735 | Muscle tone functions | SCI-SCS** | For the following health problems please rate how much of a problem it was for you in the last 3 months: Muscle spasms, spasticity |
| ICF Brief Core Set | b810 | Protective functions of the skin | SCI-SCS** | For the following health problems please rate how much of a problem it was for you in the last 3 months: Pressure sores, decubitus |
| ICF Brief Core Set | d230 | Carrying out daily routine | MDS*** | In the last 4 weeks, how much of a problem have you had carrying out daily routine? |
| ICF Brief Core Set | d240 | Handling stress and other psychological demands | MDS*** | In the last 4 weeks, how much of a problem have you had handling stress? |
| ICF Brief Core Set | d550 | Eating | SCIM**** | Independence in activities of daily living: Eating and drinking |
| ICF Brief Core Set | d520 | Caring for body parts | SCIM**** | Independence in activities of daily living: Grooming |
| ICF Brief Core Set | d530 | Toileting | SCIM**** | Independence in activities of daily living: Using the toilet |
| ICF Brief Core Set | d420 | Transferring oneself | SCIM**** | Independence in activities of daily living: Transfers from the bed to the wheelchair |
| ICF Brief Core Set | d465 | Moving around using equipment | SCIM**** | Independence in activities of daily living: Moving around moderate distances (10 to 100 meters) |
| ICF Brief Core Set | d445 | Hand and arm use | SCI-FI/AT Basic Mobility ***** | Are you able to push open a heavy door? |
| ICF Brief Core Set | d455 | Moving around | MDS*** | In the last 4 weeks, how much of a problem have you had getting where you want to go? |
| ICF Brief Core Set | d470 | Using transportation | MDS*** | In the last 4 weeks, how much of a problem have you had using public transportation? |
|  |  |  | MDS*** | In the last 4 weeks, how much of a problem have you had using private transportation? |
| ICF Brief Core Set | d410 | Changing basic body position | SCI-FI/AT Basic Mobility ***** | Are you able to get up off the floor from lying on your back? |
|  |  |  | SCI-FI/AT Basic Mobility ***** | Are you able to moving from sitting at the side of the bed to lying down on your back? |
| Statistical selection^[15]^ | d570 | Looking after one’s health | MDS*** | In the last 4 weeks, how much of a problem have you had looking after your health, eating well, exercising or taking your medicine? |
| Statistical selection^[15]^ | d660 | Assisting others | MDS*** | In the last 4 weeks, how much of a problem have you had providing care or support for others? |

* 36-Item Short Form Survey; **SCI-SCS – Spinal Cord Injury Secondary Conditions scale; ***MDS– Model Disability Survey;**** SCIM - Spinal Cord Independence Measure;**** *SCI-FI/AT - Spinal Cord Injury Functional Index for samples using Assistive Technology (SCI-FI/AT).
