# Supplementary material for: Ageing, functioning patterns and their environmental determinants in the spinal cord injury (SCI) population: A comparative analysis across eleven European countries implementing the International Spinal Cord Injury Community Survey
Source: PLoS One. 2023 Apr 20;18(4):e0284420. doi: 10.1371/journal.pone.0284420 (PMC10118153; doi:10.1371/journal.pone.0284420)
Supplement: S3 Table — (DOCX) [file pone.0284420.s006.docx]

**S6 Table. Posterior predictive p-values corresponding to the observed totals.**

| **Item** | **Description** | **1** | **2** | **3** | **4** | **5** |
| --- | --- | --- | --- | --- | --- | --- |
| 1 | Feeling full of life | 0.49 | 0.50 | 0.50 | 0.50 | 0.51 |
| 2 | Feeling depressed | 0.50 | 0.50 | 0.50 | 0.50 | 0.51 |
| 3 | Feeling tired | 0.50 | 0.50 | 0.50 | 0.50 | 0.51 |
| 4 | Bowel dysfunction | 0.49 | 0.51 |  |  |  |
| 5 | Bladder dysfunction | 0.50 | 0.50 |  |  |  |
| 6 | Sexual dysfunction | 0.49 | 0.52 |  |  |  |
| 7 | Contractures | 0.50 | 0.51 |  |  |  |
| 8 | Spasticity | 0.49 | 0.51 |  |  |  |
| 9 | Skin functions | 0.50 | 0.51 |  |  |  |
| 10 | Pain | 0.50 | 0.50 |  |  |  |
| 11 | Carry out daily routine | 0.50 | 0.51 | 0.50 |  |  |
| 12 | Handeling stress | 0.50 | 0.51 | 0.51 |  |  |
| 13 | Getting to your destination | 0.49 | 0.50 | 0.49 |  |  |
| 14 | Using public transportation | 0.50 | 0.51 |  |  |  |
| 15 | Using private transportation | 0.50 | 0.50 | 0.52 |  |  |
| 16 | Looking after your health | 0.51 | 0.50 | 0.50 |  |  |
| 17 | Providing care or support for others | 0.49 | 0.51 | 0.50 |  |  |
| 18 | Getting up off the floor from lying on your back | 0.50 | 0.50 | 0.51 |  |  |
| 19 | Push/open a heavy door | 0.50 | 0.52 | 0.50 | 0.50 | 0.50 |
| 20 | Moving from sitting to lying down | 0.49 | 0.51 | 0.50 |  |  |
| 21 | Eating&Drinking | 0.50 | 0.50 |  |  |  |
| 22 | Grooming | 0.51 | 0.50 |  |  |  |
| 23 | Toileting | 0.50 | 0.50 |  |  |  |
| 24 | Transfer bed-weelchair | 0.50 | 0.50 |  |  |  |
| 25 | Moving 10-100m | 0.49 | 0.52 |  |  |  |
