# Supplementary material for: Ageing, functioning patterns and their environmental determinants in the spinal cord injury (SCI) population: A comparative analysis across eleven European countries implementing the International Spinal Cord Injury Community Survey
Source: PLoS One. 2023 Apr 20;18(4):e0284420. doi: 10.1371/journal.pone.0284420 (PMC10118153; doi:10.1371/journal.pone.0284420)
Supplement: S4 Table — (DOCX) [file pone.0284420.s007.docx]

**S7 Table. Items' discrimination and items thresholds for all sample.**

| **Item** | **Description** | **Items' discrimination** | **Item Threshold 1** | **Item Threshold 2** | **Item Threshold 3** | **Item Threshold 4** |
| --- | --- | --- | --- | --- | --- | --- |
| 1 | Feeling full of life | 0.51 | -3.13 | 0.42 | 1.76 | 2.94 |
| 2 | Feeling depressed | 0.53 | -1.35 | 0.47 | 2.45 | 4.03 |
| 3 | Feeling tired | 0.46 | -5.10 | -2.06 | 1.82 | 3.37 |
| 4 | Bowel dysfunction | 0.69 | -1.66 |  |  |  |
| 5 | Bladder dysfunction | 0.56 | -1.50 |  |  |  |
| 6 | Sexual dysfunction | 0.43 | -2.88 |  |  |  |
| 7 | Contractures | 0.73 | -1.12 |  |  |  |
| 8 | Spasticity | 0.68 | -2.03 |  |  |  |
| 9 | Skin functions | 0.70 | 0.96 |  |  |  |
| 10 | Pain | 0.58 | -2.57 |  |  |  |
| 11 | Carry out daily routine | 1.50 | -0.80 | 1.17 |  |  |
| 12 | Handeling stress | 0.75 | -0.61 | 2.16 |  |  |
| 13 | Getting to your destination | 1.58 | -0.78 | 0.64 |  |  |
| 14 | Using public transportation | 1.73 | -0.84 |  |  |  |
| 15 | Using private transportation | 1.41 | 0.20 | 1.04 |  |  |
| 16 | Looking after your health | 1.02 | -0.01 | 1.83 |  |  |
| 17 | Providing care or support for others | 1.43 | -0.50 | 0.73 |  |  |
| 18 | Getting up off the floor from lying on your back | 1.98 | -1.30 | -0.11 |  |  |
| 19 | Push/open a heavy door | 1.56 | -0.87 | -0.13 | 0.42 | 0.94 |
| 20 | Moving from sitting to lying down | 2.17 | -0.18 | 0.81 |  |  |
| 21 | Eating&Drinking | 2.43 | 0.80 |  |  |  |
| 22 | Grooming | 2.49 | 0.60 |  |  |  |
| 23 | Toileting | 1.51 | -0.88 |  |  |  |
| 24 | Transfer bed-weelchair | 2.11 | 0.50 |  |  |  |
| 25 | Moving 10-100m | 1.08 | 0.34 |  |  |  |
