# Supplementary material for: Ageing, functioning patterns and their environmental determinants in the spinal cord injury (SCI) population: A comparative analysis across eleven European countries implementing the International Spinal Cord Injury Community Survey
Source: PLoS One. 2023 Apr 20;18(4):e0284420. doi: 10.1371/journal.pone.0284420 (PMC10118153; doi:10.1371/journal.pone.0284420)
Supplement: S1 File — (PDF) [file pone.0284420.s008.pdf]

S3 file. The Hierarchical Generalized Partial Credit Model.

In test situation with ordinal items, the response of a person i from a country g on item j is symbolized by Y_{ij}^g. For each jth item, the response is categorized as one of the K_j possibilities, ranging from 1 to K_j, with K_j=2, 3, 4, or 5 in this study. In the Generalized Partial Credit Model (GPCM) (1), the probability that the person i achieves a category on item j is given by:

$$P(Y_{ij}^g = y_{ij}|\theta_i^g, \beta_j^g, \alpha_j^g) = \frac{\exp\{\sum_{h=1}^{y_{ij}^g} \alpha_j^g(\theta_i^g - \beta_{jh}^g)\}}{\sum_{k=1}^{K_j} \exp\{\sum_{h=1}^{k} \alpha_j^g(\theta_i^g - \beta_{jh}^g)\}}, y_{ij} = 1, ..., K_j,$$

Here, θ_i^g is the ability of person i from the group g, a lower θ_i indicating low probability for reaching a higher category. The parameter vector $\beta_j^g = (\beta_{j1}^g, ..., \beta_{jK_j}^g)$ denotes the thresholds of item j. The model imposes that $\beta_{j1}^g = 0$, corresponding to the reference category 1 for each item j = 1, ...,J. The threshold indicates the probability of crossing from one category in the response to the immediate next choice (higher or lower in trait). Thus, when the thresholds are ordered in the analysis of questionnaire data, i.e. $\beta_{j_2}^g \leq \beta_{j_3}^g \leq ... \leq \beta_{jK_j}^g$, the response categories are assumed to be ordered as well. The occurrence of reversed thresholds indicates that the order of the response categories is violated. By definition, α_j^g, is the positive discrimination power of item j. A higher α_j^g favours the kth category over the *(k-1)*th category with increasing θ_i^g.

To make a common scale across groups, traditional multi-group IRT models request no Differential Item Functioning (DIF) for at least one item, used in the calibration with the other items (2). In contrast, in the hierarchical IRT implementation as described by De jong et.al. (3), there is no longer a need to classify items as being invariant or noninvariant across countries: the DIF is accommodated by using a random-effects ANOVA formulation for items thresholds as: $\beta_{jh}^g = \beta_{jh} + \epsilon_{jh}^g$, i.e. item group threshold of each item is modelled as overall mean threshold, β_{jh} plus the group-specific deviation, ϵ_{jh}^g. This approach implies to impose a hierarchical group structure in the latent scale: $\theta_i^g = \theta^g + \delta_i^g$, where δ_i^g is the individual score of student i in group g, and θ^g is group g's mean score. We have set the variances of the threshold parameters to not vary across items. The items' discriminations were considered as invariant across groups.

Prior distributions

For items parameters, we followed the previous literature recommendations (4) (5). We denoted the country mean as θ^g, with a weakly informative prior distribution, so that it doesn't have a major impact on the posterior distribution, but stabilized the model. For the identification of latent variable, the sum of each country thresholds is set to 0. This was done by forcing the sum of ϵ_j^g to be equal with 0. Table 1 shows the prior distribution for each parameter:

Appendix 1: Table 1: Prior distribution

Parameter	Prior distribution	Constraint
α_j	$\alpha_j \sim N(0, 10)$	$\alpha_j > 0$
β_j	$\beta_j \sim N(0, 5^2)$	$\sum_{j=1}^{J} \beta_j = 0$
ϵ_j^g	$\epsilon_j^g \sim N(0, 1)$	$\sum_{j=1}^{J} \epsilon_j^g = 0$
δ_i^g	$\delta_i^g \sim N(0, 1)$	
θ^g	$\theta^g \sim N(0, 10000)$	

References

[1] Muraki E: A generalized partial credit model: Application of an em algorithm. Applied Psychological Measurement 1992, 16:159-176.

[2] Holland PW, Wainer H: Differential item functioning. Hillsdale, NJ: Erlbaum, 1993.

[3] De Jong, MG, Jan-Benedict EMS, Fox JF: Relaxing Measurement Invariance in Cross-National Consumer Research Using a Hierarchical IRT Model. Journal of Consumer Research 2007; 34: 260-278.

[4] Sahu SK. Bayesian estimation and model choice in item response models. Journal of Statistical Computation and Simulation. 2002; 72, 217–232.

[5] Sinharay S, Johnson MS, Stern HS. Posterior predictive assessment of item response theory models. Applied Psychological Measurement. 2006; 30(4), 298–321.
