# Supplementary material for: Ageing, functioning patterns and their environmental determinants in the spinal cord injury (SCI) population: A comparative analysis across eleven European countries implementing the International Spinal Cord Injury Community Survey
Source: PLoS One. 2023 Apr 20;18(4):e0284420. doi: 10.1371/journal.pone.0284420 (PMC10118153; doi:10.1371/journal.pone.0284420)
Supplement: S2 File — (PDF) [file pone.0284420.s009.pdf]

S4 file. Posterior predictive p-value

Using estimated the model parameters, a new replicated data Y_{ij}^{rep}, i = 1, ...,n, j = 1, ...,J is calculated. The comparison of predictive distribution Y_{ij}^{rep} with the observed Y_{ij} is generally named as "posterior predictive check". Although the posterior predictive check involves a double-use of the data, an identification of a systematic difference may indicate a potential failing of the model. The posterior predictive distribution is calculated by MCMC as follows (1)(2):

1) for each iteration l=1,...,L, L=5000, model parameters are estimated: θ_i^l, β^l,α^l;

2) based on estimated parameters a replicated data $Y^{(rep,l)}$ is calculated;

3) for the obtained replicated data, a summary statistic $S(Y^{(rep,l)})$ is calculated, S(y), here, the total number of respondents for each of the categories of each of the 25 items:

$$S_{jk}(y) = \sum_{i=1}^{n} I(y_{ij} = k)$$

where I(A) denotes the indicator function of its argument A. The posterior predictive p-value is calculates as:

$$P(S_{jk}(y^{rep}) \geqq S_{jk}(y) \mid y)$$

A posterior predictive "p-value" of .5 means that S(y) will be exactly equal to the median of the posterior predictive distribution of $S(y^{rep})$. If our model's predictions are "biased" to be too high, then we will get a number greater than .5, and if they are generally on the low side, we will get a number less than .5.

References

[1] Sinharay, S. Assessing fit of unidimensional item response theory models using a Bayesian approach. Journal of Educational Measurement 2005, 42(4), 375–394.

[2] Sinharay, S, Johnson MS, Stern HS. Posterior predictive assessment of item response theory models. Applied Psychological Measurement 2006, 30(4), 298–321.
