# Supplementary material for: Ageing, functioning patterns and their environmental determinants in the spinal cord injury (SCI) population: A comparative analysis across eleven European countries implementing the International Spinal Cord Injury Community Survey
Source: PLoS One. 2023 Apr 20;18(4):e0284420. doi: 10.1371/journal.pone.0284420 (PMC10118153; doi:10.1371/journal.pone.0284420)
Supplement: Attachment — Submitted filename: review.docx [file pone.0284420.s010.docx]

**Ageing, functioning patterns and their environmental determinants in the Spinal Cord Injury (SCI) population: a comparative analysis across eleven European countries implementing the International Spinal Cord Injury Community Survey.**

Thank you for the opportunity to review this interesting paper! This study used data collected in eleven European countries to describe functioning patterns of the SCI population in light of chronological age, age at injury and time since injury as well as to identify environmental determinants of functioning.

Personally, I’m very impressed by the InSCI in general and this study doesn’t seem to be an exception. However, I have limited knowledge of the statistical methods used for developing the functioning metric and the PMWD technique, and this must be taken into account when reading this review.

The manuscript is well written and it seems as the work behind it is very thorough and rigorous. The authors provide a lot of background information to strengthen the methods they have used (however, I can’t seem to find additional file 2). They are transparent with limitations regarding representative data and it’s a strength that they report representative data and data from countries using convenience samples separately.

**Specific comments**
Introduction
line 93: ”a life” typo.
line 97: “growth” typo.
line 127: the authors could consider adding more information about why the aging process is important to study in the context of SCI, for example an increased vulnerability to age-related conditions and diseases, smaller margins to recover after an illness or injury etc.
Lines 152-155: As the authors write, aging with SCI is a complex process. I do not object to the fact that functioning is a key health indicator but the authors state that: “Given that the functioning indicator has the potential to capture the complexity of ageing with SCI in a single score, it is a highly relevant measure to understand the lived experience of ageing with SCI, …” The ICF conceptualizes a person's level of functioning as a dynamic interaction between her or his health conditions, environmental factors, and personal factors. In this study, the authors don’t touch upon the lack of personal factors in their functioning metric. Moreover, to “capture the complexity of ageing with SCI” and “understand the lived experience of ageing with SCI”, I believe a broader concept than functioning is needed. In my opinion there is also a need to take the persons’ subjective perspective and experiences into consideration. The ICF does not include QoL or more subjective measures of well-being, such as life satisfaction, which has been criticized. I would have liked to see a discussion about these issues (i.e., the exclusion of personal factors in the functioning metric and the lack of more subjective evaluations of the aging person’s life situation) if the authors indeed set out to use their metric to capture the complexity of aging and understand the lived experience of SCI. If not, please consider rephrasing this paragraph.

Methods
lines 204-205. Please provide more information about the InSCI, such as data collection processes, recruitment etc, maybe as a fact box or similar, or at least a reference.
lines 217-218: I would have liked to see more information about how the authors reasoned when selecting items for their functioning metric. Was there a consensus discussion, did they include people with lived experience etc? For example, why not use the ISCoS Activity and Participation Data set? And there is nothing about respiration, infections, hospitalizations or sleep to mention a few other areas of relevance for people with SCI.
There is also a need to include more information about the different assessment tools from which the authors extracted the items.

Discussion
The authors acknowledge the lack of some expected findings, such as no association between age and lower levels of functioning in persons with tetraplegia and no association between time since injury and functioning. However, I would have liked to see a more elaborate discussion about possible underlying mechanisms for these findings, or lack thereof. Could it be related to the functioning metric, the study design or something else?
Line 540: How could these barriers be addressed by policy makers?
Line 551-552: How, more specifically, do the results from this study contribute to innovations in the SCI aging agenda?
Lines 552-560: There is an ongoing longitudinal project about aging with long-term SCI in Sweden with so far 12 publications, the authors could have referenced it: Jörgensen S, Iwarsson S, Norin L, Lexell J. The Swedish Aging with Spinal Cord Injury Study (SASCIS): Methodology and initial results. PM R. 2016;8(7):667-77.
Line 497: From the abovementioned project, a recent study identifies declines in physical independence and an increase in certain secondary health conditions which could have been added to the discussion: Waller M, Jörgensen S, Lexell J. Changes over six years in secondary health conditions and activity limitations in older adults aging with long-term spinal cord injury. PM R 2022. doi: 10.1002/pmrj.12776

Conclusion
Lines 583-584: Referring back to my last comment regarding the introduction, “Having a focus how well persons with SCI live and age” seems a bit too broad for the functioning metric. I suggest the authors to rephrase in line with the wording in the conclusion in the abstract.
